# Effect of S53P4 bioactive glass and low-level laser therapy on calvarial bone repair in rats submitted to zoledronic acid therapy

**DOI:** 10.1590/ACB360603

**Published:** 2021-07-09

**Authors:** Caio Peres Bellato, Danilo Louzada de Oliveira, Marcus Vinicius Satoru Kasaya, David Moreira, Marcelo Augusto Cini, Patricia Pinto Saraiva, Jéssica Lemos Gulinelli, Pâmela Leticia Santos

**Affiliations:** 1 Fellow PhD degree. Postgraduate Program in Oral and Maxillofacial Surgery. Assistant Professor. Department Oral and Maxillofacial Surgery – Dental School – Universidade do Oeste Paulista – Presidente Prudente (SP), Brazil.; 2 PhD, Assistant Professor. Oral and Maxillofacial Surgery – Department Oral and Maxillofacial Surgery – Dental School – Universidade do Oeste Paulista – Presidente Prudene (SP), Brazil.; 3 PhD. Oral and Maxillofacial Surgery – Department of Postgraduate – Dental School – Centro Universitário Sagrado Coração – Bauru (SP), Brazil.; 4 PhD. Oral and Maxillofacial Surgery – Department of Postgraduate – Dental School – Centro Universitário Sagrado Coração – Bauru (SP), Brazil.; 5 PhD, Assistant Professor. Basic Science – Oral Biology – Universidade do Oeste Paulista – Jau (SP), Brazil.; 6 PhD, Assistant Professor. Oral and Maxillofacial Surgery – Oral Sin – Londrina (PR), Brazil.; 7 PhD, Assistant Professor. Oral and Maxillofacial Surgery – Department of Health Sciences – Dental School – Universidade de Araraquara – Araraquara (SP), Brazil.

**Keywords:** Disphosphonates, Lasers, Bone Regeneration, Biocompatible Materials, Rats

## Abstract

**Purpose:**

To evaluate the influence of bioactive glass and photobiomodulation therapy (PBMT) in calvarial bone repair process in rats submitted to zoledronic acid therapy.

**Methods:**

Twenty-four rats were selected and treated with the dose of 0.035 mg/kg of zoledronic acid every two weeks, totalizing eight weeks, to induce osteonecrosis. After the drug therapy, surgical procedure was performed to create 5-mm diameter parietal bone defects in the calvarial region. The rats were then randomly assigned to groups according to the following treatments: AZC: control group, treated with blood clot; AZBIO: bone defect filled with bioactive glass; AZL: treated with blood clot and submitted to PBMT; and AZBIOL: treated with bioactive glass S53P4 and submitted to PBMT. Tissue samples were collected and submitted to histomorphometric analysis after 14 and 28 days.

**Results:**

At 14 days, bone neoformation in the AZBIO (52.15 ± 9.77) and AZBIOL (49.77 ± 13.58) groups presented higher values (p ≤ 0.001) compared to the AZC (23.35 ± 10.15) and AZL groups (23.32 ± 8.75). At 28 days, AZBIO (80.24 ± 5.41)still presented significant higher bone recovery values when compared to AZC (59.59 ± 16.92)and AZL (45.25 ± 5.41) groups (p = 0.048). In the 28-day period, the AZBIOL group didn’t show statistically significant difference with the other groups (71.79 ± 29.38).

**Conclusions:**

The bioactive glass is an effective protocol to stimulate bone neoformation in critical defects surgically created in rats with drug induced osteonecrosis, in the studied periods of 14 and 28 days.

## Introduction

Bone regeneration is a complex process consisting of the activation of various biological responses, including cellular and molecular mechanisms. This process is essential for bones to resume their usual functions of load bearing, mobility, protection, hematopoiesis, and endocrine homeostasis^1^,^2^. In critical bone defects, this process becomes even more difficult, because the regeneration cannot occur spontaneously, and, when resulting from trauma, tumor resection and congenital malformations, can become a problem in reconstructive bone surgery^3^. In addition, there is another aggravating factor, that is the medications used for low bone mass or cancer metastasis to bone tissue, retarding the bone restoring process after craniofacial reconstructive surgeries. Thus, it is important to use materials allocated within these defects and all existing resources, as these will induce and lead to bone neoformation to its full extent^4^.

The S53P4 bioactive glass (BonAlive Biomaterials, Turku, Finland) is used as a bone substitute biomass for autogenous bone. It is composed of silica and a mixture of oxides (53% SiO_2_, 23% Na_2_O, 20% CaO and 4% P_2_O_5_). Due to its osteoconductive, angiogenic and antibacterial properties, this material is recognized for promoting satisfactory bone regeneration^5^,^6^.

Photobiomodulation therapy (PBMT) is a noninvasive modality, also assists in the bone repair process and it is currently considered the main alternative treatment, because it stimulates angiogenesis, accelerates particle resorption within bone defects and increases osteoblastic activity^7^-^10^. Moreover, studies showed that the PBMT stimulates the mitochondrial and cellular membrane photoreceptors to synthetize of adenosine triphosphate (ATP), which enhances cell proliferation rate^11^, increasing the proliferation and differentiation of osteoblasts^12^, and in soft and hard tissue surgeries improves and accelerates healing^13^,^14^.

However, in addition to critical defects, bone repair capacity can be adversely affected by drugs that act to cause osteoclast apoptosis, preventing bone turnover from occurring and, consequently, delaying the bone recovery process after craniofacial and orthopedic reconstructive surgery^3^,^9^. Among these drugs, bisphosphonates are commonly used to prevent and treat increased bone resorption in skeletal diseases and are classified as antiresorptive drugs. Zoledronic acid is a third generation bisphosphonate, with a high action potential used as a therapeutic agent for conditions associated with malignant neoplasms, and present medicated osteonecrosis as side effect and complications^15^-^18^.

Considering the admittedly positive influence of S53P4 bioactive glass and PBMT on the bone repair process, this study aimed to analyze the bone recovery of critically defects created in the calvarial bones of rats with drug osteonecrosis induced by zoledronic acid, through histomorphometric analysis.

## Methods

The project was submitted and approved by the Animal Use Ethics Committee (CEUA) of the Centro Universitário Sagrado Coração (Unisagrado), Bauru, SP, Brazil, filed under CEUA no. 4974081216.

Twenty-four female rats (*Rattus norvegicus albinus*, Wistar), weighing approximately 300 grams, from the Unisagrado bioterium, were used^19^. The animals were kept in polypropylene cages, lined with white autoclaved pine wood shavings changed three times a week. Throughout the experimental period, the animals remained in the bioterium of the Unisagrado, under controlled environmental conditions of temperature (22 ± 2ºC), adequate ventilation, with dark/light cycle of 12 hours each, receiving ordinary feed and water without restriction.

### Zoledronic acid treatment

**Figure 1:**
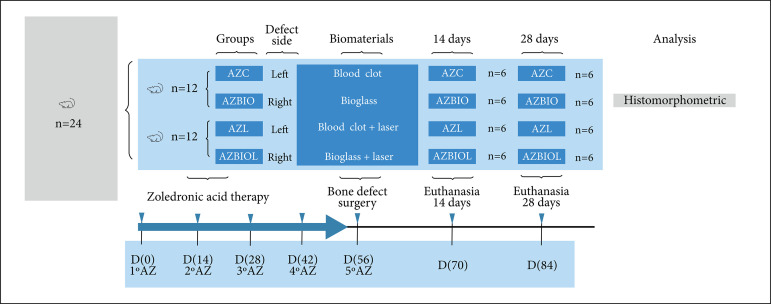
Flowchart exemplifying intravenous zoledronic acid therapy and group division.

The animals underwent intravenous zoledronic acid therapy (Zometa, Novartis Pharma AG, Basel, Switzerland) at a dosage of 0.035 mg/kg administered once every two weeks for an eight-week period^9^. Fifty-six days after the beginning of drug therapy, the surgical procedure was performed to create bone defects in the calvarial region of all animals^19^. The subjects were randomly distributed into four groups (n = 6):

- AZC group: bone defect filled with blood clot (control group);
- AZBIO group: bone defect filled with bioactive glass S53P4;
- AZL group: bone defect filled with blood clot and submitted to PBMT;
- AZBIOL group: bone defect filled with bioactive glass S53P4 and submitted to PBMT (Fig. 1).

### Surgical procedure

All subjects were sedated by intramuscular injection of anesthetic with 1% ketamine at a dose of 50 mg/kg (Francotar, Virbac, São Paulo, SP, Brazil) associated with the sedative 2% xylazine hydrochloride at a dose of 5 mg/kg (Virbaxyl, Virbac, São Paulo, SP, Brazil), in accordance with the dosage recommended by the manufacturer.

The trichotomy of the parietal bone region and the antisepsis with 2% chlorhexidine (Riohex, Rioquímica, São José do Rio Preto, SP, Brazil) was performed in the regions selected to be incised. After antisepsis, the animals received a local anesthetic with 0.3 mL/kg 2% mepivacaine hydrochloride (scandicaine with 1:100,000 epinephrine, Septodont, Paris, France), intended to promote trans-surgical hemostasis and immediate transoperative and postoperative analgesia.

A skull-caudal longitudinal incision of approximately 20-mm length was made into the subjects’ scalp over the sagittal suture of the calvarial region using a no.-15 carbon steel blade (Feather Industries, Tokyo, Japan) mounted on a no.-3 scalpel (Hu-Friedy, Frankfurt, Germany). The soft tissue was detached and removed with the aid of delicate syndesmotomes (Quinelato, Rio Claro, SP, Brazil), allowing access to the cortical bone in the respective region of the parietal bones. Subsequently, bi-cortically defects were made surgically, creating two bone defects of 5 mm in diameter, laterally distributed to the median sagittal suture of the parietal bones, using a 5-mm trephine drill (Neodent, Curitiba, PR, Brazil) coupled to the contra-angle (NSK SG20, 20:1, Tokyo, Japan) on an electric implant motor (NSK Surgic XT Plus, Tokyo, Japan), at the speed of 800 rpm under copious irrigation with 0.9% physiological solution throughout the ostectomy, to avoid thermal necrosis of bone tissue.

After surgery, the bone defects were instantly filled with blood clot or bioactive glass S53P4, according to the group treatment that they were distributed. The animals from groups AZL and AZBIOL (PBMT group) were then immediately submitted to PBMT with aluminum gallium arsenide (ArAlGa) (Photon Laser, DMC Equipamentos, São Carlos, SP, Brazil), with a wavelength of 660 nm, spot size of 0.07 cm^2^, power of 0.03 W, during 133 seconds per point, irradiance of 0.42 W/cm^2^, and energy of 4 J/point (57.14 J/cm^2^/point). Each region received a total energy of 32 J. The applications were performed in the central region and at seven points on the periphery of the bone defect, totaling eight application points. The beam diameter was 4 ± 1 mm, and the probe was placed in contact with the bone tissue^9^.

Following the treatment procedures, the soft tissues were carefully repositioned and sutured with monofilament suture (Nylon 5.0, Ethicon, Johnson & Johnson, São José dos Campos, Brazil) with simple interrupted stitches, leading to primary wound closure, then the antisepsis of the region with 2% chlorhexidine (Riohex, Rioquímica, São José do Rio Preto, SP, Brazil, Brazil) was once again performed to remove blood residues.

In the immediate postoperative period, the animals received a single administration of the antibiotic Flotril 2.5% (Schering-Plough, Rio de Janeiro, RJ, Brazil) at a dose of 0.2 mL/kg, and analgesic dipyrone Analgex (Agener União, Sao Paulo, SP, Brazil) at a dose of 0.06 mL/kg, by intramuscular applications. Analgesic application was maintained every 12 hours for three days. No feeding or movement restrictions were imposed to the animals, which were kept inside the cages throughout the experiment.

After the periods of 14 and 28 days, the animals were euthanized intraperitoneally with 1% ketamine overdose (Francotar, Virbac, São Paulo, SP, Brazil) for samples removal.

### Histological processing

Calvarial bone specimens containing the bone defect region were removed and immersed in 10% buffered neutral formalin (Bio-Optica, Milano, Italy) for 48 hours, then decalcified in 5% ethylenediaminetetraacetic acid (EDTA) (Titriplex III, Merck, Darmstadt, Germany) for 20 days, with two exchanges within 10 days. After the descaling process was completed, the samples were washed in running water for 24 hours, dehydrated and diaphanized. In the plastic state, a microtome slide was made using a section in the central portion of each bone defect, following an imaginary line that crosses its central portion in anteroposterior direction, thus dividing each bone defect into two equal halves. The pieces were processed according to paraffin embedding protocol (Histosec, Merck, Darmstadt, Germany) and hematoxylin and eosin (HE) staining in addition to Masson’s trichrome (MT).

Three contiguous 5-μm thick histological sections were made on a microtome (Leica RM 2145, Leica Biosystems, Nussloch, Germany) from the median longitudinal section, sequentially placed on a glass slide that was coded so that the observer remained unaware of the details regarding thedistribution and characteristics of the study groups.The histological analysis comprehended the full extent of the critical defect, using the x40 magnification for the observation of the inflammatory infiltrate and the x20 magnification for the analysis of the bone repair process, with a resolution of 300 dpi under a binocular microscope, totaling five regions in each sample.

### Morphological analysis

The following aspects were considered in the morphological evaluation: inflammatory infiltrate pattern, presence or absence of biomaterial particles and newly formed bone tissue. Images were taken using a 1.3-megapixel resolution image capture camera (Leica DFC 300FX, Leica Microsystems, Heerbrugg, Switzerland) coupled with a visible light microscope and a computer (Leica Aristoplan Microsystems, Leitz, Benshein, Germany).

### Histometric analysis

Histological samples were stained with MT for the histometric analysis. The images were analyzed at a 20x magnification, and the formation of bone tissue present in the bone defect region was quantified using the ImageJ program. According to Garcia *et al*.^19^, the total area to be analyzed corresponded to the entire area of the original surgical defect. This area was determined by first identifying the external and internal surfaces of the original calvarium at the right and left margins of the surgical defect, and then connecting them with lines drawn following their respective curvatures. Considering the total length of the histologic specimen, a distance of 2 mm was measured from the right and left edges of the specimen towards the center in order to determine the margins of the original surgical defect. The newly formed bone area was delineated within the confines of the total area. The total area was measured in square millimeters and was considered to represent 100% of the area to be analyzed. The newly formed bone area was also measured in square millimeters and calculated as a percentage of the total area.

After obtaining the histometric values, the analyzed groups were coded, and the data was submitted to the Kolmogorov-Smirnov statistical test to investigate possible intra and intergroup differences, as well as to verify their normality. It was observed that the measured data were non-parametric, so the Mann-Whitney statistical test at a significance level of 5% was used.

The statistical tests employed in this study were Kruskal-Wallis and analysis of variance (ANOVA), complemented by the Student-Newman-Keuls test. All statistical tests were evaluated at a significance level of 5% (p < 0.05) using the SigmaStat 3.2 program.

## Results

### Morphological analysis

The results of the morphological qualitative analysis were obtained through the assessment of the following bone defect structures: defect borders, morphology of newly formed bone tissue, connective tissue characteristics (cell arrangement and organization), type of inflammatory infiltrate and remnant bone replacement after 14 and 28 postoperative days.

**Figure 2:**
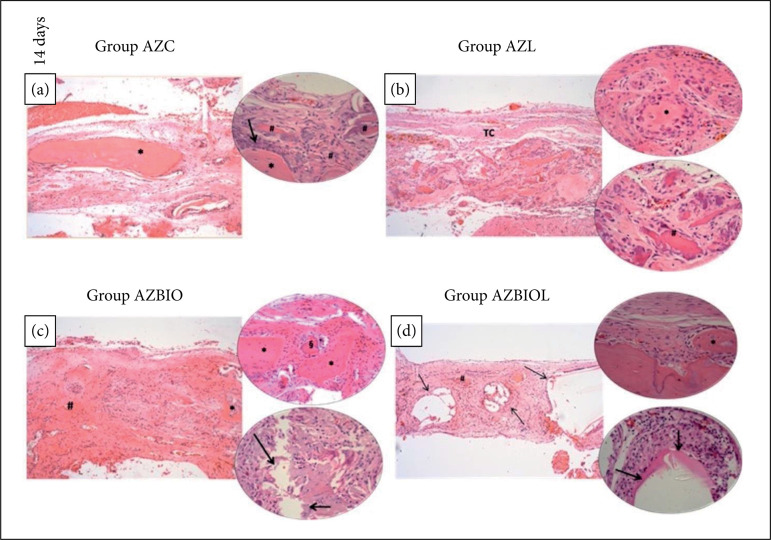
Photomicrographs of sections stained with hematoxylin and eosin in post-operative periods of 14 days: **(a)** Central region of bone defect filled with granulation tissue (*gt*), collagen fibers, non-viable fragments of bone tissue (*) and bone neoformation at the margins of the defect (*#*); **(b)** Bone neoformation in the margins of the defect (*). In the central region, there is the presence of granulation tissue (*gt*) and of fragments of non-viable bone tissue (*#*), surrounded by giant cells (*arrows*); **(c)** Central area of the defect with intense inflammatory infiltrate (*ii*), formed by predominantly mononuclear cells (§) bordering biomaterial particles (*) and fragments of non-viable bone tissue (*#*). Presence of large amount of giant cells around the non-viable bone tissue (*arrows*); **(d)** Presence of biomaterial in the central area of the defect (*arrows*), permeated by connective tissue (*CT*) and fragments of necrotic bone tissue (*). Original magnification of x10 and x20.

**Figure 3:**
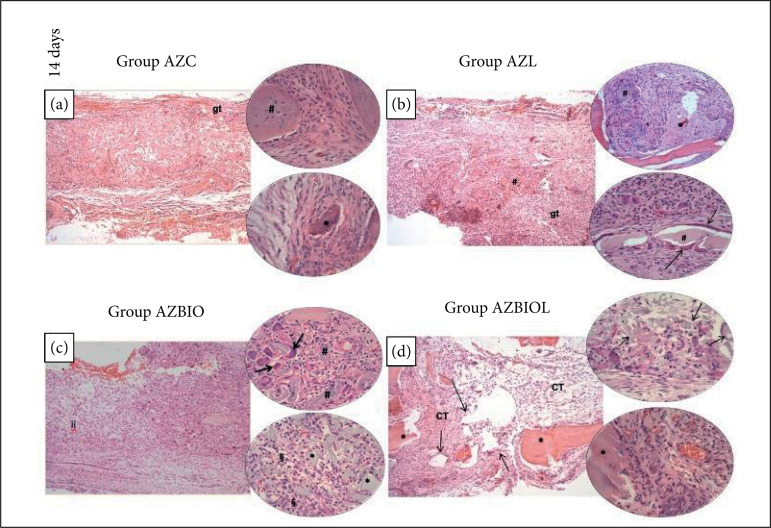
Photomicrographs of sections stained with hematoxylin and eosin in post-operative periods of 28 days: **(a)** Bone neoformation in the marginal region of the defect (*), bordered by osteoblastic cells (*arrow*), in connective tissue and nonviable bone particles (*#*); **(b)** Central region of the defect filled with connective tissue (*CT*), few areas of bone neoformation (*) and fragments of nonviable bone tissue (*#*); **(c)** Central region of bone defect filled with fibrous connective tissue (*#*). Presence of neoformed bone tissue (*) in the margin of the bone defect and adjacent areas and presence of necrotic bone (§). More centralized regions containing biomaterial (*arrows*); **(d)** Bone neoformation in areas near the margin of the defect (*). Central region containing fragments of biomaterial (*arrows*) and non-viable bone tissue (*#*). Original magnification of x10 and x20.

In the AZC group (study control group), after 14 days, neoformed bone tissue was observed at the extremities of the defect and in the central region, presenting a granulation tissue containing a large amount of blood vessels, fibroblasts, predominantly mononuclear inflammatory infiltrate, few polymorphonuclear (PMN) and giant cells around non-viable bone fragments (Fig. 2a). After 28 days, a higher bone neoformation in the defect extremities with reduced number of osteocytes in the central region was observed, including the presence of connective tissue with predominant mononuclear infiltrate cells, few PMN and some giant cells (Fig. 3a).

Regarding the AZL group (PBMT), after 14 days, newly formed bone tissue was observed in the defect extremities, while in the central region a richly vascularized and cellularized granulation tissue was observed, with predominance of mononuclear inflammatory infiltrate, moderate number of PMNs and few giant cells bordering non-viable bone fragments (Fig. 2b). After 28 days, it was possible to observe a connective tissue occupying the central region of the defect with predominant mononuclear inflammatory cells, few PMN and giant cells around non-viable bone tissue (Fig. 3b).

In the AZBIO group (S53P4 bioactive glass), after 14 days, it was observed bone neoformation in the extremities and central region of the defect, with the presence of biomaterial particles, predominant mononuclear inflammatory infiltrate cells, moderate number of PMN, a large number of giant cells around non-viable bone fragments and few others bordering the biomaterial (Fig. 2c). After 28 days, bone neoformation continued to be observed in the extremities and central region of the defect, with the presence of biomaterial encapsulated by connective tissue, infiltrate containing predominance of mononuclear cells, moderate number of PMNs and giant cells around non-viable bone tissue (Fig. 3c).

In the AZBIOL group (S53P4 bioactive glass and PBMT), after 14 days, bone neoformation was observed in the extremities and central region of the defect, containing some particles of the biomaterial surrounded by connective tissue capsules, presenting predominantly mononuclear inflammatory infiltrate cells and moderate number of PMNs (Fig. 2d). After 28 days, larger particles of the biomaterial encapsulated by connective tissue were noted, with predominance of mononuclear cells, moderate number of PMNs and giant cells around non-viable bone fragments (Fig. 3d).

### Histometric analysis

A statistically significant difference was observed comparing the AZC (p = 0.003), AZL (p = 0.005) and AZBIO (p ≤ 0.001) groups over time. As for the AZBIOL group, there was no statistically significant difference between the 14 and 28-day periods (p = 0.167).

**Figure 4:**
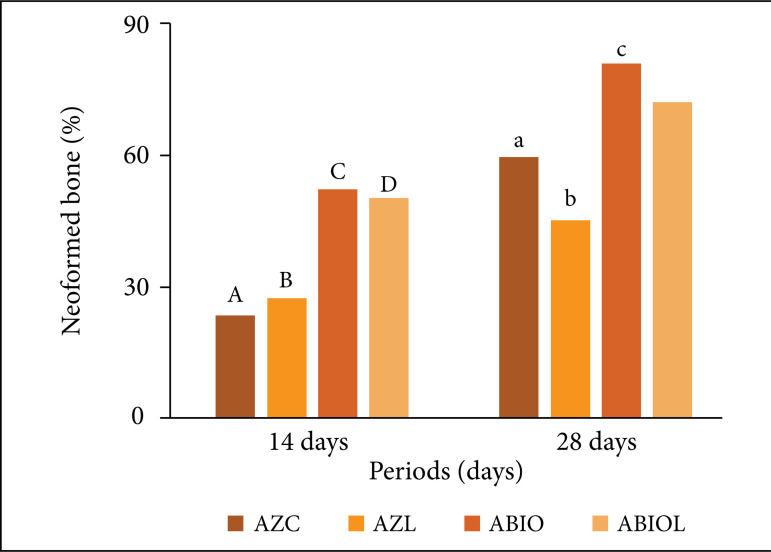
Graphic representation of means of histometric analysis related to bone neoformation area in percentage. Statistically significant difference within the group: a-A (p = 0.003); b-B (p = 0.005) and c-C (p ≤ 0.001). In the intergroup evaluation, a statistically significant difference: 14 days: C-A; C-B; D-A; D-B (p ≤ 0.001).28 days: c-a; c-b (P = 0.048).

After the 14-day period, a statistically significant difference between the groups (p ≤ 0.001) was observed in the intergroup evaluation. The AZBIO (52.15 ± 9.77) and AZBIOL (49.77 ± 13.58) groups showed higher bone formation compared to the AZC (23.35 ± 10.15) and AZL (23.32 ± 8.75) groups. After the 28-day period, the comparison of the intergroup analysis also showed a statistical difference (p = 0.048), with the AZBIO group (80.24 ± 5.41) presenting higher bone recovery than the AZC (59.59 ± 16.92) and AZL groups (45.25 ± 5.41).In 28-day period, the AZBIOL group didn’t show statistically significant difference with the other groups (71.79 ± 29.38) (Fig. 4).

## Discussion

The process of bone repair in critical defects is limited because it cannot occur spontaneously, and, in patients undergoing bisphosphonate drug treatments, this prognosis becomes even more unfavorable, susceptible to drug osteonecrosis^20^. However, low-power laser assists in the bone repair process, as S53P4 bioactive glass does, due to its osteoconductive, angiogenic and antibacterial properties^5^,^6^. Given the scarcity of studies evaluating the combined action of laser therapies and bioactive glass materials as bone substitutes in the bone repair process in a zoledronic acid-induced osteonecrosis scenario, this study aimed to evaluate through histomorphometric analysis the effect of PBMT and S53P4 bioactive glass in the process of bone repair of critical bone defects created in the calvaria of rats with induced drug osteonecrosis, showing an effective therapy in these conditions.

Studies show an induction of osteonecrosis in these animals submitted to zoledronic acid treatment in experimental models by using five biweekly applications of intravenous 0.035 mg/kg dose in the caudal vein of the rats^9^,^26^,^27^. This has also be seen in this study, since all the animals submitted to zoledronic acid application demonstrated histological characteristics of non-viable, acellular bone fragments in the presence of mono or polymorphonuclear inflammatory infiltrate throughout the defect, after 14 and/or 28 days of the surgical procedure, consistent with the histological characteristics of drug osteonecrosis^28^. Hellstein^29^ described microscopic features of osteochemonecrosis: the majority bone nonvital without evidence of osteocytes within individual lacunae; vital bone showed osteocyte present and osteoclasts may be seen within Howship lacunae (giant osteoclasts—large numbers of nuclei with cells—are uncommon).

Zoledronic acid is a third generation intravenous bisphosphonate, the most potent available clinically and used frequently in the treatment of metastatic bone lesions. It inhibits bone resorption through direct and indirect effects on osteoclasts, which undergo apoptosis or become unable to differentiate from hematopoietic stem cells. A potential side effect of this medication is osteonecrosis of the jaws, reported by Marx, which presents between 5 and 10% of patients with cancer applied with zoledronic acid^30^-^32^.

When used in bone repair process, the PBMT provides bio-stimulation, accelerating osteocyte metabolism and stimulating mesenchymal cells to differentiate into osteoblasts, increased ATP and alkaline phosphatase (ALP) concentration and the release of calcium, and promotes a specific stimulus to osteogenesis during the initial stages of healing. With this, there is an increase in the release ofhydroxyapatite crystals that will favor maturation of newly formed bone tissue, being also helpful in treating osteonecrosis lesions^33^,^34^. However, there is no consensus on the laser application protocol to be used, as showed in Table 1 35-38. In the present study, PBMT was used with a wavelength of 660 nm, made on a single application in the immediate postoperative period, thinking of minimizing discomfort when application occurs in human patients and positively influence bone repair^9^. Furthermore, in this study we chose this wavelength because of the scarcity of studies carried out with such a methodology, thus requiring greater scientific proof.

**Table 1:** Studies that evaluated bone repair with photobiomodulation therapy.

Author (year)	Defect region (rat)	Laser	Laser application method	Evaluated periods	Analysis	Conclusion
Barushka *et al.* 35	Tibia	Gallium-aluminum-arsenide λ = 808 nm	Five and six days postoperatively once a day for 2 minutes	Nine, 10, 11, 12 and 15 days	Histologic and Histomorphometric	The laser favored the repair of fractures or acute defects in the bones
Ninomiya; Ozawa^36^	Femur	Gallium-aluminum-arsenide λ = 1,064 nm	10 minutes, twice a day	One, three, five and seven days	Histologic and Histomorphometric	The laser increased in bone volume, trabecular thickness, mineral apposition rate, bone mineral density index
Kazancioglu *et al*.^21^	Calvaria	Gallium-aluminum-arsenide λ = 808 nm	120 seconds a day, three days a week, for two weeks	One month	Histologic and Histomorphometric	The laser increased bone formation compared to the control group
Yildirimturk *et al*.^37^	Tibia	Gallium-aluminum-arsenide λ = 820 nm	Three times a week for four weeks	Four weeks	Histologic	Beneficial effects on the healing of bone defects in diabetic conditions
de Oliveira *et al*.^25^	Calvaria	Gallium-aluminum-arsenide λ = 780 nm	Immediately after surgery and at intervals of 48 and 96 hours	21 and 30 days	Radiographic, Histological, Immunohistochemistry and Immunofluorescence	Favored bone repair
Pinheiro e*t al*.^38^	Tibia	Gallium-aluminum-arsenide λ = 780 nm	48-hour intervals for two weeks	15 and 30 days	Spectroscopy	It improved the repair of bone defects grafted with the biomaterial by increasing the deposition of hydroxyapatite phosphate as marked by biochemical estimators
Atasoy *et al*.^34^	Tibia	Gallium-aluminum-arsenide λ = 940 nm	Immediately after suturing, two, four, six, eight, 10 and12 days after	Four and eight weeks	Histopathological	It may not accelerate the bone repair process in the early and late stages compared to the control group without laser application
Dereci *et al*.^23^	Calvaria	Gallium-aluminum-arsenide λ = 980 nm	5 minutes immediatelyafter surgeryand six days after	21 days	Histomorphometric	Significantly increased bone regeneration in critical defects when compared to the control group
Bosco *et al*.^22^	Calvaria	Gallium-aluminum-arsenide λ = 660 nm	Eight points around the defect and a central point immediately making the defect	30 and 60 days	Histologic and Histomorphometric	Improved bone repair and accelerated the resorption of biomaterial particles
Moreira *et al*.^25^	Calvaria	Gallium-aluminum-arsenide λ = 780 nm	Four points around and one in the center, only once	30 days	Histomorphometric	There was no increase in bone neoformation when associated with autogenous bone or bioactive glass

λ: wavelength.

Low-power laser applications have angiogenic properties, accelerate particle resorption within bone defects and increase osteoblastic and osteoclastic activities^7^,^8^. These same characteristics of angiogenesis pattern modification were observed in this study, related to the treatment of the PBMT in AZL group after 28-day period, since a statistically difference in the increased number of blood vessels was seen. As for the AZBIOL group (S53P4 bioactive glass and PBMT), there was no significant difference, since the bone recover parameters remained similar to the other groups after 14-day and 28-day period.

Although the laser has as its main property the stimulation of increased angiogenesis that would favor bone neoformation, in the AZBIOL group the effects weren’t statistically significant because it is a critical defect. This fact was proven both in the qualitative and quantitative analysis. In none of the specimens the total defect closed, justifying the ability of the laser to be an adjuvant in the process of repairing critical defects and not having an inductive or osteogenic property. If there is no matrix or framework to induce repair, as in the case of insertionof the biomaterial (bioactive glass), which has the property of osteoconduction and the ability to serve as an environment for the infiltration of undifferentiated mesenchymal cells, osteoblasts and osteoclasts, acting as a passive framework that is slowly absorbed and replaced in the bone repair process, neoformation does not occur. There must be a framework with a structure and porosity similar to the one of the native bone trabecular, so that the laser action can exist, the proliferation of vessels can occur and cause the complete closure of the defect. However, when associated with the S53P4 bioactive glass, a higher bone formation was observed in both periods of 14 and 28 days.

The selection of the S53P4 bioactive glass in this study was due to its antibacterial and angiogenic property that differs from other bone substitutes. The bioactive material is composed of several ionic compounds (SiO_2_, Na_2_O, CaO, P_2_O_5_) which when implanted to the receptor surface releases alkaline ions, cause an abrupt pH increase, thus allowing inhibition of bacterial growth through osmotic and acid-base imbalance generated in the local, not being dependent on local antibiotics applications^39^,^40^. The osteoconductive capacity of bioactive glass has been demonstrated in several previous scientific studies (Table 2) 41-48, which is consistent with our research data, since it was observed a statistically significant difference in bone neoformation after 14-day and 28-day period in the groups filled with the bioactive glass (submitted or not to PBMT) when compared with the control group AZC.

**Table 2:** Studies that used bioactive glass as a bone substitute.

Author (year)	Experimental model/defect region	Defect size	Euthanasia	Use of membrane	Analysis	Conclusion
Zazgyva *et al*.^41^	Rabbit femur	4 mm	Five weeks	No	Histologic	Early start of a bone repair process
Camargo *et al*.^42^	Rabbit femur	1 cm	Two weeks	No	Histomorphometric	Bioactive glass, when used to fill cavity defects in rabbits, shows superiority in the number of osteoblasts and inferiority in the number of osteocytes when compared to the autograft
Gunn *et al*.^43^	Rat femur	4,5 mm	Three, six, 12 and 24 weeks	No	Histomorphometric	S53P4 glass can be considered a better bone filling than coral-derived calcium carbonate
Tolli *et al*.^44^	Rat femur	8 mm	Eight and 10 weeks	No	Radiographic, tomographic and mechanical	Bioglass granules appear to perform well as a bone protein extract carrier
Tuusa *et al*.^45^	Rabbit frontal bone	5 mm	Three, six and eight weeks	No	Histomorphometric	The new bone formation was occasionally seen as small spots in close contact with the polymer surface and the bioactive glass granule
Aho *et al*.^46^	Rabbit tibia	10 mm	Four and eigth weeks	No	Histomorphometric and radiographic	Bone substitute that can be used in the reconstruction of major defects
Narhi *et al*.^47^	Rabbit tibia	4 mm	Eight and 16 weeks	No	Histomorphometric	Glass granules can only conduct bone growth efficiently, as long as a direct contact between the glass and the bone can be achieved. The biocompatibility of the composite should be better by improving the contact of the glass granules with the surrounding bone, creating porous interconnected structures within the composite or accelerating the rate of degradation of the copolymer matrix
Aho *et al*.^48^	Rabbit femur	6 mm	Four, eight and 23 weeks	No	Scanning electron microscopy	It has been shown to satisfy several of the properties required for an ideal injectable bone, being bioactive and biocompatible, osteoconductive, ductile and conveniently injectable with short hardening time

## Conclusions

Based on the experimental methodology used in this study, it can be concluded the S53P4 bioactive glass is an effective protocol to stimulate bone neoformation in critical defects surgically created in rats with drug induced osteonecrosis, in the studied periods of 14 and 28 days. However, it is necessary further research on the use of photobiomodulation associated with bioactive glass in critical bone defects to investigate the best biological mechanisms during the phases that make up the complete repair process, with similar protocols and with the same defect diameter, to help determine the therapeutic window and the best treatment protocol for this type of injury.
